# Unregulated Peptide Use in the Age of Biohacking: Digital Promotion, Gray-Market Access, and Emerging Public Health Risks

**DOI:** 10.7759/cureus.110657

**Published:** 2026-06-11

**Authors:** Kirubel T Hailu, Feven N Abriha, Yeabsera M Duguma, Ryan R Haddad, Tewodros Liyew, Alousious Kasagga

**Affiliations:** 1 Public Health, University College Cork, Cork, IRL; 2 Internal Medicine, Richmond University Medical Center, Staten Island, USA; 3 Medicine, Jimma University Medical School, Jimma, ETH; 4 Surgery, Kadisco General Hospital, Addis Ababa, ETH; 5 Clinical Research, California Institute of Behavioral Neurosciences and Psychology, Fairfield, USA; 6 Public Health, Washington University in St. Louis, St. Louis, USA; 7 Pathology, Peking University, Beijing, CHN

**Keywords:** biohacking, digital health communications, peptides, pharmacovigilance, regulatory agencies, regulatory science, self-experimentation

## Abstract

Unregulated peptide use is emerging as a digitally mediated public health concern. Although peptide-based medicines have important therapeutic roles when developed, prescribed, manufactured, and monitored through regulated pathways, online biohacking and wellness spaces increasingly promote experimental or weakly evidenced peptides for fat loss, recovery, aesthetics, cognition, performance, and longevity. This narrative review examines how digital promotion, gray-market access, self-injection, stacking, informal titration, product-quality uncertainty, regulatory ambiguity, and weak pharmacovigilance interact to normalize poorly traceable peptide products outside clinical supervision. The central concern is not legitimate peptide medicine, but consumer experimentation with products of uncertain identity, purity, potency, sterility, and safety. Improved clinician awareness, adverse-event reporting, product-quality monitoring, digital risk communication, and proportionate regulatory oversight are needed to distinguish evidence-based peptide therapy from unregulated consumer use.

## Introduction and background

Peptides are short chains of amino acids that can function as hormones, signaling molecules, receptor ligands, or therapeutic agents [1,2]. In clinical medicine, peptide-based drugs occupy an important space between small-molecule pharmaceuticals and larger biologics, often offering biological specificity while remaining adaptable through chemical modification [1,3]. Although current public interest often presents peptides as a new frontier in health optimization, peptide therapeutics are not new. Since the introduction of insulin, peptide-based medicines have become established across metabolic, endocrine, reproductive, infectious disease, oncologic, and sexual health indications [1,4].

The recent expansion of peptide-based medicines has been especially visible in metabolic disease. GLP-1 receptor agonists and related incretin-based therapies have reshaped the treatment of type 2 diabetes and obesity, with agents such as semaglutide and tirzepatide demonstrating substantial effects on body weight and cardiometabolic outcomes in clinical trials [5-8]. Other peptide-based agents, including tesamorelin for HIV-associated lipodystrophy and bremelanotide for acquired, generalized hypoactive sexual desire disorder in premenopausal women, further illustrate the broader therapeutic legitimacy of this drug class [4,9]. These examples show that peptides are not inherently fringe or speculative. When developed, evaluated, manufactured, prescribed, and monitored through regulated pathways, peptide-based medicines can be clinically valuable interventions [1-3].

However, the visibility of approved peptide therapies has coincided with rising nonclinical peptide experimentation. The public success of GLP-1 receptor agonists has made injectable metabolic therapies more visible and normalized in public discourse, particularly for goals related to weight, metabolism, and body composition [5,8,10]. This legitimacy appears to be spilling over into a less regulated consumer marketplace, where experimental, investigational, or weakly evidenced peptides are promoted for fat loss, injury recovery, longevity, cognition, libido, aesthetics, sleep, muscle gain, and performance enhancement [10-12]. The concern is not that all peptides are equivalent, but that digital discourse and online access pathways may blur distinctions between approved medicines, compounded products, investigational drugs, research chemicals, context-specific clinical therapies, preclinical compounds, and products supported mainly by anecdote or marketing [10-13].

This blurring is amplified by biohacking culture, longevity medicine, wellness clinics, and influencer-led health optimization. Biohacking and do-it-yourself biology communities have been shaped partly through online forums and digital spaces where users exchange knowledge, techniques, protocols, and interpretations of biological self-experimentation [14]. In peptide-related discussions, compounds are frequently framed as tools for optimization, recovery, performance, aesthetics, cognition, or longevity, with terms such as "stacks," "protocols," "research peptides," "tier lists," and "longevity peptides" creating a shared language of self-experimentation [15]. These digital sources should not be interpreted as evidence of clinical efficacy or safety. However, they are relevant to public health because online creators, forums, and wellness communities can influence how health products are perceived, normalized, sourced, combined, and interpreted outside formal clinical supervision [15,16].

Gray-market access is central to this exposure pathway. Many peptide products are sold online with disclaimers such as "for research use only" or "not for human consumption," while being discussed in consumer spaces as substances for personal use [11,12]. Consumers may obtain peptides through direct-to-consumer websites, research-chemical vendors, informal online communities, international suppliers, compounding-related pathways, wellness or testosterone clinics, and social media-linked sellers [11-13]. Certificates of analysis, purity claims, and biomedical language may provide reassurance, even when products have not undergone the regulatory review, manufacturing oversight, or postmarketing surveillance expected for approved medicines [11,12]. In this gray-market environment, access may appear medically adjacent while remaining weakly accountable, poorly traceable, and difficult to monitor through conventional pharmacovigilance systems [11-13].

The public health concern, therefore, extends beyond whether any single peptide is effective or harmful. Unregulated peptide use creates risk through multiple interacting pathways: uncertain product identity, variable purity, inaccurate potency, contamination, nonsterility, degraded compounds, unclear storage conditions, dosing uncertainty, reconstitution errors, self-injection, stacking, drug co-use, regulatory ambiguity, and weak adverse-event surveillance [17-19]. These risks are intensified when biologically active products are used chronically, combined with prescription drugs, anabolic agents, testosterone, supplements, or GLP-1 medications, or administered by populations not studied in clinical trials [12,15,18]. They may also be intensified when dosing decisions, adverse-effect interpretation, or product selection are guided by online communities rather than clinical monitoring [15].

A further concern relates to supply-chain integrity. The most immediate risks in gray-market peptide use are likely to involve accidental or commercial failures, including mislabeling, contamination, inaccurate potency, nonsterility, counterfeit products, unstable formulations, poor manufacturing conditions, or harmful impurities [20,21]. However, the same opacity that permits poor-quality or falsified products could also create theoretical vulnerabilities to deliberate adulteration or malicious substitution [20,22]. Direct evidence that gray-market peptide products are being used as vehicles for deliberate biological harm remains limited. Nevertheless, the normalization of self-injected, poorly traceable biological products warrants attention as part of a broader supply-chain governance framework, particularly as regulators and biosecurity experts increasingly emphasize traceability, screening, and oversight in biotechnology supply chains [20,22].

Taken together, these concerns show that unregulated peptide use should be understood not simply as individual risk-taking or isolated product misuse, but as a digitally mediated public-health exposure shaped by therapeutic legitimacy, online promotion, gray-market access, consumer self-experimentation, product-quality uncertainty, regulatory ambiguity, supply-chain opacity, and incomplete surveillance [12,15,17-19]. Existing pharmacovigilance systems may be poorly positioned to detect these exposures because products may be undisclosed, mislabeled, sourced through multiple vendors, or difficult to link causally to adverse events; this concern is consistent with broader evidence that voluntary adverse-event reporting systems are vulnerable to underreporting and attribution barriers [23,24]. This narrative review examines how these forces interact to normalize poorly traceable peptide products outside conventional clinical, regulatory, and pharmacovigilance systems.

The central problem is not legitimate peptide medicine but the growing consumer use of peptide products outside accountable clinical, manufacturing, and pharmacovigilance systems. In online biohacking and wellness markets, these products may be accessed or self-administered despite limited assurance about their quality, source, dosing, and safety.

This narrative review examines how therapeutic legitimacy, digital promotion, gray-market access, consumer self-experimentation, product-quality uncertainty, regulatory ambiguity, supply-chain opacity, and weak surveillance contribute to poorly traceable peptide use outside conventional clinical and regulatory systems.

## Review

Methods

This narrative review used a structured narrative approach to synthesize peer-reviewed literature, regulatory sources, and selected digital gray literature on unregulated peptide use as an emerging public health exposure. A narrative approach was selected because the topic spans clinical pharmacology, public health, digital health communication, regulatory science, consumer behavior, biohacking culture, gray-market supply chains, and biosecurity. Narrative reviews are appropriate when the aim is to integrate heterogeneous evidence, clarify concepts, and develop interpretive frameworks rather than produce pooled effect estimates [25,26]. The objective was not to estimate prevalence, efficacy, or harm quantitatively, but to integrate diverse sources and characterize the evolving landscape of nonclinical peptide use.

Peer-reviewed literature was searched using PubMed, Scopus, Web of Science, and Google Scholar from database inception to April 29, 2026, which was treated as the final data-extraction date. Relevant sources included clinical trials, pharmacological reviews, regulatory analyses, case reports, pharmacovigilance studies, toxicology reports, product-quality studies, public health commentaries, and digital health research. Gray literature was searched from regulatory and public health sources, including the United States Food and Drug Administration (FDA), European Medicines Agency (EMA), World Health Organization (WHO), World Anti-Doping Agency (WADA), national drug regulators, poison-center reports, clinical trial registries, regulatory warning letters, and high-quality media investigations. Gray-literature searching is particularly useful in public health topics where relevant evidence may be produced by regulators, public agencies, professional bodies, or other nonjournal sources [27,28]. Regulatory information was interpreted as current as of April 29, 2026.

Digital media sources were reviewed to characterize how peptides are promoted, discussed, and normalized online. These included YouTube videos, podcasts, online clinician commentary, influencer content, consumer forums, media investigations, and biohacking or wellness communities. Digital sources were not treated as evidence of clinical efficacy or safety, but as gray-literature material reflecting public discourse, marketing narratives, consumer motivations, sourcing pathways, self-experimentation practices, and risk perception. Vendor websites and affiliate-linked content were reviewed for promotional claims and access pathways, but not as independent user discourse. Single-anecdote reports from social media or forums were not treated as representative of population-level patterns.

Evidence was interpreted in relation to the type of claim being made. Sources based on clinical or regulatory evidence were used when discussing established therapeutic use, while regulatory alerts, pharmacovigilance material, and product-quality evidence were used to support concerns about safety, oversight, and market practices. Digital sources were included to understand how peptides are promoted, accessed, and discussed by consumers, but they were not treated as evidence of clinical efficacy or safety.

Search terms combined peptide-specific, behavioral, regulatory, and digital-market concepts, including "unregulated peptides," "peptide misuse," "biohacking peptides," "research use only peptides," "peptide gray market," "direct-to-consumer peptides," "peptide self-injection," and "peptide stacking." Searches also included commonly discussed compounds such as BPC-157, TB-500, CJC-1295, ipamorelin, GHK-Cu, MOTS-c, epitalon, melanotan, semax, selank, and retatrutide. Additional targeted searches addressed regulatory and market-related terms, including "FDA peptide warning letter," "unapproved GLP-1 products," "compounded semaglutide," "research use only drugs," "retatrutide warning," and "bulk drug substances compounding."

**Figure 1:**
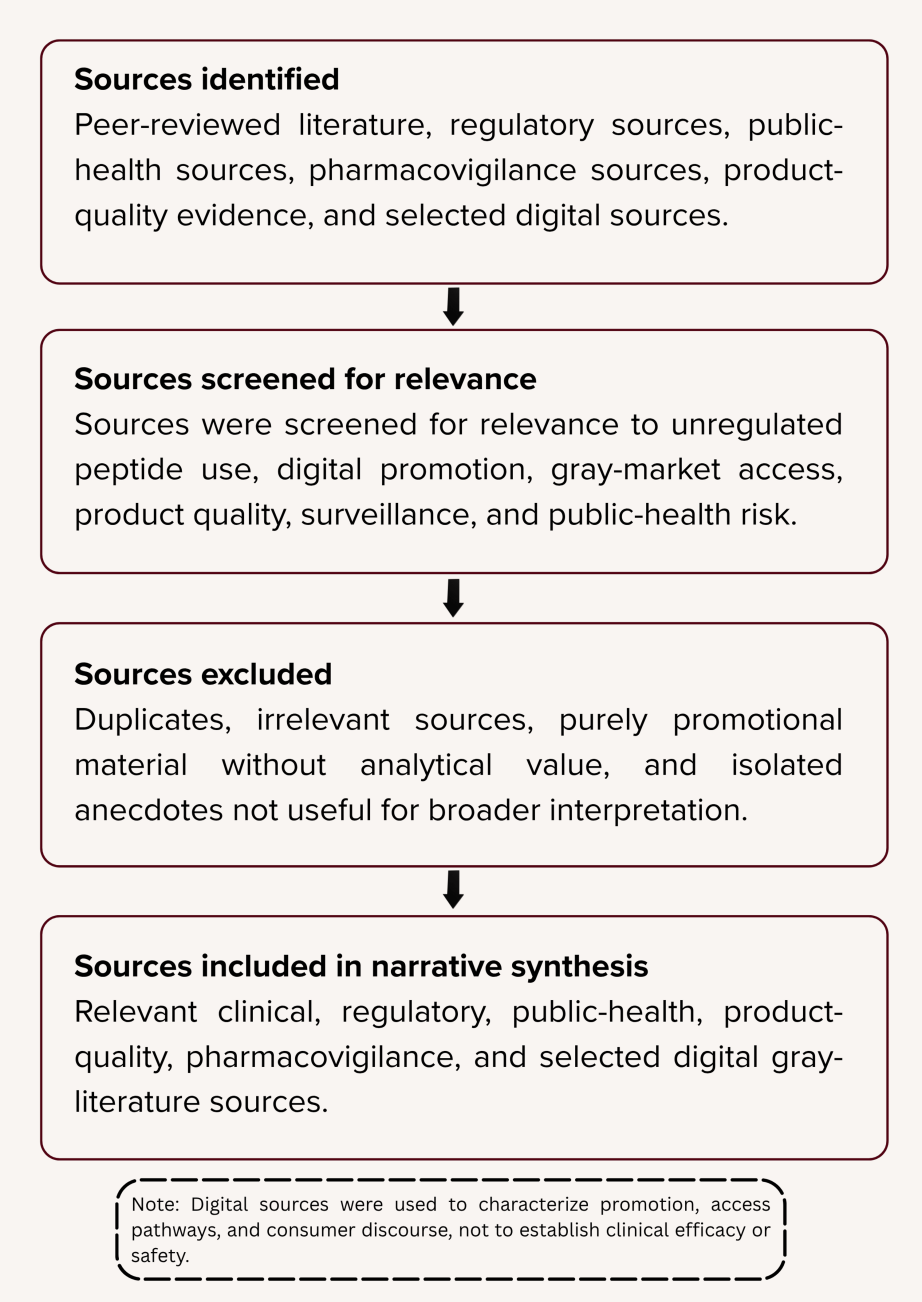
Source identification and selection process for the narrative review Flowchart showing how sources were identified, screened, excluded, and included in the narrative synthesis. Digital sources were used to characterize promotion, access pathways, and consumer discourse, not to establish clinical efficacy or safety. Original figure created by Kirubel T. Hailu using Canva (Canva Pty Ltd., Sydney, Australia). No artificial intelligence assistance was used in the creation of this image.

Evidence was classified into four categories: approved therapeutic use supported by clinical trials and regulatory review; clinically used but indication- or population-specific evidence; limited human evidence, preclinical evidence, or mechanistic plausibility; and primarily anecdotal, promotional, or digital-discourse evidence. This hierarchy was used to avoid inappropriate generalization from regulated therapeutic success to unapproved or weakly evidenced consumer use. The source identification and selection process is summarized in Figure 1.

Limitations of this review

Digital discourse sources were sampled rather than systematically scraped or quantified; gray-market access pathways are opaque; adverse-event data may capture only severe, acute, or recognized events; and regulatory information may change rapidly, particularly regarding compounding policy, enforcement actions, and the legal status of specific peptide products.

Review of literature

Conceptual Framework: The Digital Peptide Exposure Pathway

**Figure 2:**
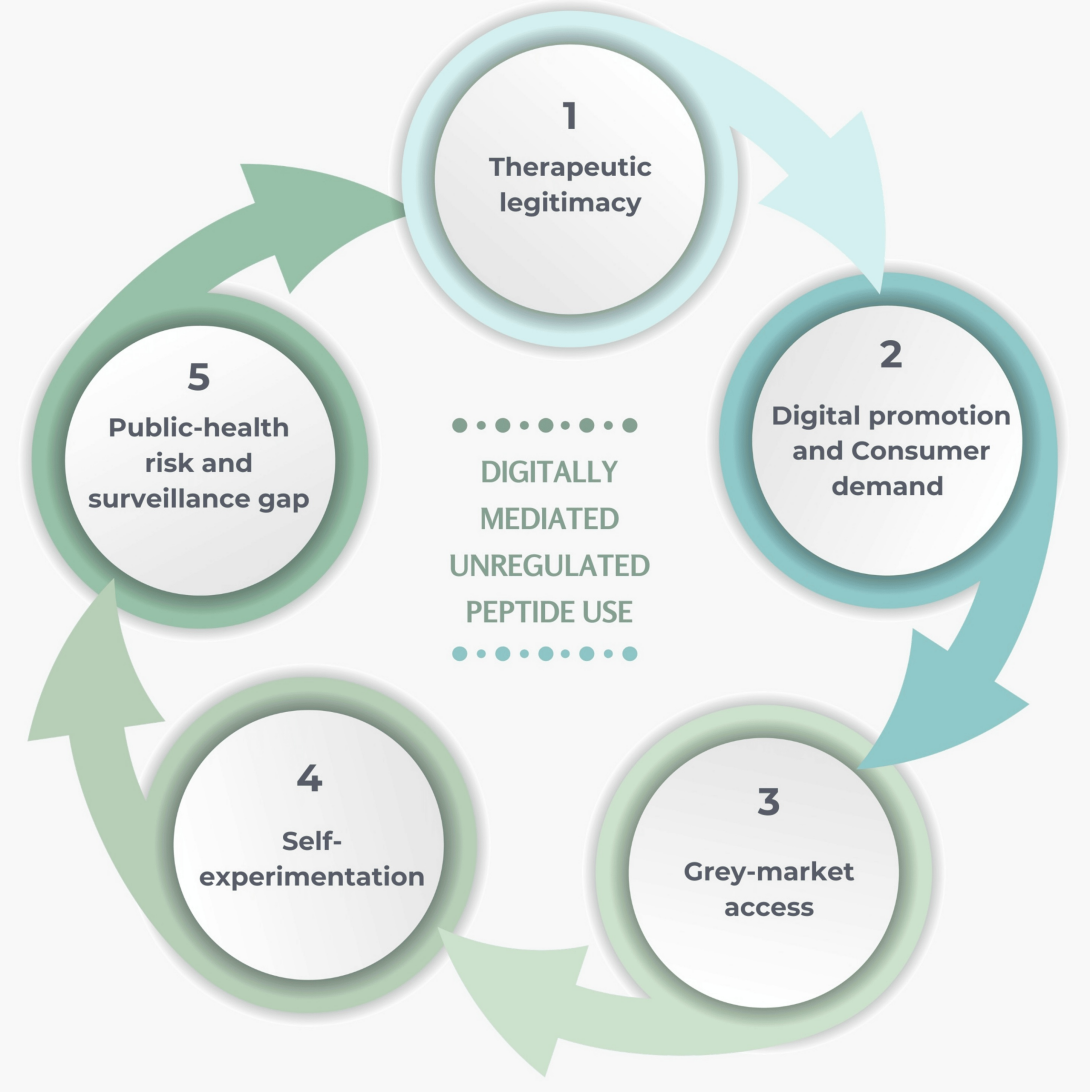
The digital peptide exposure pathway Conceptual framework showing how approved peptide therapies may influence digital promotion, consumer demand, gray-market access, self-experimentation, and public health surveillance gaps. Sources: [1,5,7,10-12,14,15,20-24,29]. Original figure created by Kirubel T. Hailu using Canva (Canva Pty Ltd., Sydney, Australia). No artificial intelligence assistance was used in the creation of this image.

This review conceptualizes unregulated peptide use as a digitally mediated exposure pathway rather than a conventional medication-use pattern. In regulated care, peptide-based medicines are prescribed for defined indications, manufactured under quality standards, dispensed through authorized channels, and monitored through clinical follow-up and pharmacovigilance. In contrast, unregulated peptide use often emerges through a pathway in which therapeutic legitimacy, digital promotion, consumer demand, gray-market access, and self-experimentation interact to produce poorly monitored exposure. Figure 2 summarizes the proposed digital peptide exposure pathway. 

Therapeutic legitimacy derived from approved peptide medicines may be amplified through digital promotion, generating consumer demand and facilitating gray-market access. This may lead to self-experimentation with poorly traceable products, creating product-quality, biological, and public health risks that remain incompletely captured by existing surveillance systems.

The pathway begins with the clinical success of regulated peptide medicines. Insulin anchors the long history of peptide therapeutics, while newer agents such as semaglutide and tirzepatide illustrate the recent visibility of peptide-based treatment in metabolic disease [1,5,7]. Tesamorelin and bremelanotide further show that peptide-based medicines have legitimate clinical roles beyond diabetes and obesity [4,9]. However, this legitimacy may be transferred in public discourse to experimental or weakly evidenced peptide products, leading consumers to interpret the term "peptide" as implying medical credibility, biological sophistication, or relative safety.

Digital platforms amplify this perceived legitimacy. Biohacking communities have historically developed through online spaces where users exchange biological self-experimentation knowledge and practices [14]. In peptide-related discussions, forums and online communities may frame peptides as tools for recovery, optimization, performance, aesthetics, cognition, or longevity rather than as pharmacologically active compounds requiring careful evaluation [15]. Social media health content can also influence how medicines and health products are interpreted by the public [10,16].

Consumer demand is then translated into exposure through gray-market pathways. Direct-to-consumer websites, research-chemical vendors, online clinics, informal sellers, international suppliers, social media-linked vendors, and online communities may all contribute to access outside conventional clinical channels [11,12,29]. Products may be labeled "for research use only" or "not for human consumption," while still being discussed in ways that suggest self-administration [11,12]. Once obtained, consumers may self-inject, stack, cycle, titrate informally, combine peptides with other agents, and use online communities to interpret effects or adverse symptoms [15,17].

Risk arises from overlapping product-related, biological, behavioral, regulatory, and supply-chain domains. Product-related risks include uncertain identity, inaccurate potency, contamination, nonsterility, degradation, and unreliable quality claims [12,21]. These concerns overlap with broader risks associated with substandard or falsified medical products, particularly when supply chains are informal, online, or poorly traceable [20]. Biological risks arise because peptides may influence endocrine, immune, metabolic, reproductive, growth-factor, or tissue-repair pathways when used outside studied populations or without monitoring. Supply-chain opacity also raises concerns about mislabeling, counterfeit products, and, more theoretically, deliberate adulteration or malicious substitution [20,22].

The final stage is surveillance failure. Existing systems may poorly capture unregulated peptide exposure because users may not disclose use, clinicians may not ask about it, products may be mislabeled, and adverse events may be attributed to other causes. These problems are consistent with broader limitations of voluntary adverse-event reporting systems, including underreporting and difficulty attributing harms to poorly characterized products [23,24]. This framework, therefore, positions unregulated peptide use as a systems-level public health issue: a convergence of therapeutic innovation, digital promotion, commercial incentives, gray-market access, consumer self-experimentation, weak supply-chain oversight, and incomplete monitoring.

Evidence and Digital Claim Landscape

A central challenge in evaluating unregulated peptide use is that the term "peptide" is applied across very different clinical, regulatory, and consumer contexts. Online discourse often places approved medicines, investigational drugs, compounded products, research chemicals, and anecdotal wellness peptides within the same category. This can create confusion for consumers and complicate public health analysis. For this reason, this review distinguishes peptide products by regulatory status, evidence strength, and context of use.

At one end are established therapeutic peptides with defined indications, regulated manufacturing, clinical monitoring, and evidence from clinical trials or established practice. These products demonstrate that peptide therapeutics can be clinically valuable when used within appropriate medical and regulatory systems [1,4,5,7]. A second group includes peptides with evidence in specific clinical populations or indications, but whose findings may not generalize to healthy consumers, athletes, wellness users, or people using products for optimization [4,9]. A third group includes investigational peptides that may have promising trial data but have not completed regulatory review for general consumer use; retatrutide is a prominent example because phase 2 trial results generated substantial attention while the drug remains investigational [30]. A fourth group includes experimental or wellness peptides promoted online despite limited, inconsistent, or nongeneralizable human evidence. This group includes compounds such as BPC-157, CJC-1295, ipamorelin, GHK-Cu, MOTS-c, semax, selank, and TB-500, several of which have been flagged by regulators or discussed in the literature as areas of uncertain safety, limited evidence, or nonapproved use [21,31,32]. Table 1 summarizes these tiers. 

**Table 1:** Evidence and regulatory classification of peptide use This table classifies peptide use according to regulatory status, strength of supporting evidence, and context of use. Examples are illustrative rather than exhaustive and do not imply equivalent safety, efficacy, legality, or clinical readiness across categories. Categories may overlap when approved peptide medicines enter unauthorized or poorly traceable access pathways. BPC-157, body protection compound-157; TB-500, thymosin beta-4 fragment; CJC-1295, growth hormone-releasing hormone analog; GHK-Cu, glycyl-L-histidyl-L-lysine copper complex; MOTS-c, mitochondrial open reading frame of the 12S rRNA-c. Original table created by Kirubel T. Hailu. Sources: [1,4,5,7,9,11,12,21,30-32].

Tier	Examples	Main concern
Established therapeutic medicines	Insulin; semaglutide; tirzepatide; tesamorelin; bremelanotide [1,4,5,7,9].	Therapeutic legitimacy may spill over to unrelated or weakly evidenced products [1,5,7].
Context-specific clinical use	Tesamorelin; bremelanotide; selected endocrine and reproductive peptides [4,9].	Narrow clinical evidence may be generalized to broad wellness claims [4,9].
Investigational and pipeline peptides	Retatrutide; other pipeline metabolic agents [30]	Trial visibility may be mistaken for consumer readiness or safety [30].
Experimental and wellness peptides	BPC-157; TB-500; CJC-1295; ipamorelin; GHK-Cu; MOTS-c; epitalon; semax; selank [21,31,32].	Online visibility may exceed human evidence and safety characterization [21,31,32].
Research-chemical and gray-market products	Products sold as “research use only” or “not for human consumption” [11,12,21].	Identity, purity, sterility, legality, and accountability may be unclear [11,12,21].

These categories may overlap in practice. An approved peptide medicine can become a gray-market exposure when a product with the same or a similar name is obtained through unauthorized or poorly traceable channels. The concern is therefore not legitimate peptide medicine, but peptide use outside accountable clinical and supply-chain systems.

This evidence gradient matters because online promotion can transform uncertain or preliminary evidence into confident consumer-facing claims. In metabolic disease, the clinical success of semaglutide and tirzepatide has been accompanied by direct-to-consumer interest in compounded or nonapproved GLP-1-related products, while investigational agents such as retatrutide may attract public attention before regulatory approval [29,30]. In recovery and musculoskeletal spaces, BPC-157 and related peptides are often discussed for tissue repair or injury healing, although the human evidence base remains limited relative to the strength of consumer-facing claims [15,31]. In performance-oriented spaces, growth hormone secretagogues and related compounds overlap with endocrine manipulation and anti-doping concerns [18,32]. In aesthetic and tanning spaces, melanotan-type peptides have been discussed in online forums and regulatory warnings, illustrating how peptide-like products can circulate through digital communities for cosmetic goals [12,33]. Table 2 summarizes the main digital claim categories and their associated evidence concerns. 

**Table 2:** Digital claim typology and evidence concerns This table summarizes common digital claim categories associated with peptide products and corresponding public health concerns. Examples are illustrative rather than exhaustive and do not imply equivalent safety, efficacy, legality, or strength of evidence. AOD-9604, synthetic analogue of the lipolytic domain of human growth hormone; BPC-157, body protection compound-157; TB-500, thymosin beta-4 fragment; CJC-1295, growth hormone-releasing hormone analog; GHRP, growth hormone-releasing peptide; IGF-1, insulin-like growth factor 1; GHK-Cu, copper complex of glycyl-L-histidyl-L-lysine; MOTS-c, mitochondrial open reading frame of the 12S rRNA-c; PT-141, bremelanotide. Original table created by Kirubel T. Hailu. Sources: [4,5,7,9,11,12,15,18,29-33].

Claim category	Common examples	Public health concern
Fat loss and metabolic optimization	Semaglutide; tirzepatide; retatrutide; AOD-9604 [5,7,29,30].	Spillover from approved obesity treatment to unapproved or gray-market metabolic products [11,13,29].
Healing and recovery	BPC-157; TB-500; GHK-Cu; thymosin-related peptides [15,31,33].	Injury self-treatment, unsupervised injection, and weak human evidence [15,31,33].
Muscle, performance, and body composition	CJC-1295; ipamorelin; GHRP-type peptides; IGF-1-related products [18,32].	Endocrine manipulation, stacking, anti-doping overlap, and inadequate monitoring [18,32].
Aesthetics, skin, and tanning	GHK-Cu; melanotan-type peptides [12,33].	Cosmetic normalization of systemic or injectable products [12,33].
Sexual and reproductive health	PT-141, bremelanotide; kisspeptin; melanotan-type products [4,9,12].	Broad consumer use may delay evaluation of underlying clinical conditions [4,9,12].
Cognition and mood	Semax; selank [15,33].	Self-management of neuropsychological symptoms without robust evidence [15,33].
Longevity and anti-aging	Epitalon; MOTS-c; GHK-Cu; thymosin-related peptides [15,33].	Chronic use may occur ahead of long-term human safety data [15,33].

Across these categories, the recurring concern is the mismatch between high digital visibility and limited or nongeneralizable human evidence. Technical language may be mistaken for clinical authority, certificates of analysis for regulatory assurance, and personal testimonials for generalizable evidence [10,15]. This is especially concerning when products are injected, stacked, used chronically, combined with other enhancement-oriented agents, or obtained through poorly traceable supply chains [12,21].

A structured evidence and claim landscape is therefore essential. Approved peptide medicines should not be stigmatized because of gray-market products, but neither should the success of approved medicines be used to legitimize experimental or unregulated consumer use. Public health analysis should distinguish clinical benefit under regulated conditions from investigational promise, context-specific evidence, and poorly monitored consumer experimentation.

Consumer Practices, Access Pathways, and Supply Chains

Unregulated peptide use is not limited to single-product exposure. In digital wellness and biohacking spaces, peptides are often discussed as customizable tools that can be combined, adjusted, cycled, sourced, and interpreted through personal experimentation [15,33]. Netnographic research on peptide-related forum discussions shows how digital spaces can support shared "folk pharmacology," community knowledge exchange, sourcing discussions, harm-reduction advice, and concerns about ineffective, substituted, or poor-quality products [33]. This differs from conventional medication use, where treatment is guided by diagnosis, indication, dosing standards, contraindication screening, dispensing controls, monitoring, and adverse-event reporting. Consumer peptide use may instead be shaped by online protocols, influencer narratives, peer advice, vendor materials, informal sourcing networks, and perceived bodily feedback [15,33].

One prominent practice is stacking, in which users combine multiple peptides or related products to pursue several goals simultaneously, such as weight loss, recovery, sleep, body composition, libido, cognition, or aesthetics. Stacking reflects the way peptides are marketed as modular interventions, but it creates substantial uncertainty. When several biologically active compounds are used together, it becomes difficult to identify which product is responsible for perceived benefits, adverse effects, laboratory abnormalities, or delayed complications [15,33].

A second practice is informal titration, where users adjust timing, frequency, duration, or dose pattern based on perceived effects rather than clinical guidance. Online discussions may encourage users to interpret hunger, sleep, soreness, libido, mood, skin changes, gastrointestinal symptoms, or weight fluctuations as signals for adjustment [15,33]. Although self-monitoring can appear sophisticated, subjective responses are vulnerable to placebo effects, expectation, confirmation bias, and misattribution. Symptom-based adjustment may also fail to detect silent risks such as endocrine disruption, glucose dysregulation, inflammatory responses, contamination-related effects, or organ-specific adverse events.

A third pattern is route experimentation, especially movement from oral or topical products to injectable forms. Injection may be perceived as more potent or biologically effective, but it substantially changes the risk profile by introducing concerns about sterility, reconstitution technique, dosing accuracy, injection-site infection, abscess formation, needle safety, and systemic exposure [12,17]. A product with limited or different risk in topical or regulated clinical use cannot automatically be assumed to be safe when injected in an unregulated setting.

Access pathways are equally important. Experimental or weakly evidenced peptides may circulate through direct-to-consumer websites, research-chemical vendors, online clinics, longevity or testosterone clinics, informal sellers, international suppliers, social media-linked vendors, private messaging platforms, and consumer communities [29,33,34]. The key issue is not that all nontraditional access pathways carry the same level of risk, but that current systems do not reliably distinguish legitimate clinical use, questionable wellness prescribing, research-chemical sales, counterfeit or misbranded products, and informal gray-market distribution. Products labeled "for research use only" or "not for human consumption" may still be discussed in ways that suggest self-administration [11,12,35]. For some consumers, such labels may function more as legal disclaimers than meaningful safety warnings, especially when paired with purity claims, testimonials, dosing discussions, or medically framed explanations.

Direct-to-consumer websites often use biomedical language, mechanisms of action, certificates of analysis, purity percentages, accreditation claims, or "third-party testing" claims to create an impression of legitimacy. Evidence from direct-to-consumer compounded GLP-1 markets shows how online providers may advertise compounded or nonapproved products in ways that blur clinical access, commercial promotion, and regulatory ambiguity [29,36]. In one cross-sectional study of websites selling compounded GLP-1 receptor agonists, many sites provided incomplete or potentially misleading information about compounding status, FDA approval, safety information, and efficacy claims [36]. However, certificates of analysis and purity claims may not reliably indicate pharmaceutical-grade quality, sterility, endotoxin burden, degradation, storage conditions, batch consistency, or whether the delivered product matches what was tested [12,21,36]. Clinic-like channels can also be ambiguous: some operate within legitimate clinical frameworks, while others use medical branding to promote interventions with limited evidence or unclear indications.

Informal digital communities further shape access. Forums, social media groups, Discord servers, Reddit discussions, encrypted messaging platforms, and video comment sections may function as spaces where users exchange vendor recommendations, shipping advice, product impressions, side-effect interpretations, and protocol modifications [15,33]. These peer-based trust systems may reduce friction between curiosity and purchase, but they cannot substitute for pharmaceutical quality assurance or clinical monitoring.

International supply chains complicate traceability and accountability. Peptides may be synthesized, packaged, relabeled, tested, or shipped across multiple jurisdictions before reaching consumers. A product marketed as domestic may rely on imported raw materials or overseas manufacturing, while international products may be repackaged by intermediaries. Consumers may have limited ability to verify origin, manufacturing conditions, storage history, or chain of custody. These uncertainties are especially important for injectable products, where safety depends not only on the named compound but also on sterility, concentration accuracy, solvent quality, storage, and handling [20,36].

These practices and access pathways also weaken surveillance. When products are obtained through informal vendors, research-chemical websites, international suppliers, or multiple sources, adverse events may be difficult to trace to a specific manufacturer, batch, compound, or contaminant. Users may not disclose peptide use because they do not consider peptides to be medications, are uncertain about legality, or fear judgment from clinicians. Clinicians may then attribute nonspecific symptoms to other causes, and adverse-event systems may fail to detect emerging patterns [23,24]. Pharmacovigilance research on compounded GLP-1 receptor agonists illustrates how compounded or nonstandard products can raise additional concerns around adverse events, medication errors, and product-quality reporting [35].

For public health, product risk is shaped not only by the molecule itself, but also by how it is sourced, combined, administered, monitored, and interpreted. Understanding these consumer practices, access pathways, and supply chains is therefore essential for surveillance, regulation, clinician guidance, digital risk communication, and harm-reduction strategies.

Risk, Regulation, Surveillance, and Ethical Implications

The risks associated with unregulated peptide use are multidimensional. They arise not only from the pharmacological properties of a given peptide, but also from product quality, route of administration, user behavior, supply-chain opacity, regulatory ambiguity, and weak surveillance. A peptide product may be risky because it is biologically active, contaminated, mislabeled, injected without sterile technique, combined with other agents, or poorly captured by adverse-event systems. The principal risk domains are summarized in Table 3.

**Table 3:** Risk taxonomy for unregulated peptide use This table summarizes major product-related, route-of-use, pharmacological, behavioral, surveillance, regulatory, ethical, equity, supply-chain, and biosecurity risks associated with unregulated peptide use. Risk domains are illustrative rather than exhaustive and may overlap in practice. GH, growth hormone; IGF-1, insulin-like growth factor 1. Original table created by Kirubel T. Hailu. Sources: [1,11,12,15,16,18,20-24,32,34-39].

Risk domain	Examples	Why it matters
Product-quality risks	Mislabeling, incorrect dose, wrong ingredient, impurities, degradation, contamination, non-sterility, endotoxin contamination, unreliable certificates of analysis [12,20,21,36,37].	Consumers and clinicians may not know what product was actually used [20,21,36].
Route-of-use risks	Self-injection, reconstitution errors, dosing errors, needle misuse, abscess, cellulitis, and lack of sterile technique [17,38].	Injection magnifies risks from contamination, inaccurate concentration, and poor handling [17,38].
Pharmacological risks	Endocrine disruption, GH or IGF-1 pathway activation, glucose dysregulation, immune reactions, reproductive-axis effects, pigmentation changes, drug–peptide interactions [1,18,32].	Biological activity may have unintended effects outside studied populations or combinations [1,18,32].
Behavioral risks	Stacking, informal titration, cycling, delayed care, reliance on influencer advice, and use despite contraindications [15,33].	Consumer behavior can increase exposure uncertainty and delay clinical evaluation [15,33].
Surveillance risks	Non-disclosure, clinicians not asking, vague product names, multiple vendors, low adverse-event reporting [23,24,34,39].	Harms may remain invisible to pharmacovigilance and public-health systems [23,24,34,39].
Regulatory risks	Research-use-only labeling, online sales, cross-border supply chains, compounding ambiguity, and anti-doping overlap [11,12,18,21].	Existing regulatory systems may not map cleanly onto the digital peptide marketplace [11,12,18,21].
Ethical and equity risks	Conflicts of interest, affiliate marketing, weak informed consent, stratified access to safer sourcing or monitoring [16,35].	Risk and accountability may be shifted onto consumers unequally [16,35].
Supply-chain and biosecurity risks	Counterfeit products, opaque provenance, malicious substitution or adulteration as theoretical risks [20,22,36].	Poorly traceable injectable biological products create broader governance concerns [20,22,36].

Product-quality risk is among the most immediate concerns. In regulated pharmaceutical manufacturing and sterile compounding, identity, purity, potency, sterility, stability, and batch consistency are subject to formal quality-control expectations. Standards for compounded sterile preparations are intended to reduce contamination, infection, and patient harm [37]. In unregulated or poorly regulated markets, these safeguards may be absent, inconsistent, or difficult to verify. A product labeled as a specific peptide may contain an incorrect dose, a degraded compound, impurities, bacterial or endotoxin contamination, or a different ingredient from what is listed [21,36]. Certificates of analysis may reassure consumers, but they may not be independent, batch-specific, or sufficient to assess sterility, degradation, solvent quality, storage stability, or whether the tested sample matches the product received [12,36].

Route-of-use risk is especially important because many peptides discussed in wellness, bodybuilding, longevity, and biohacking spaces are injected outside clinical settings. Self-injection introduces risks separate from the peptide's biological activity, including injection-site inflammation, abscess, cellulitis, dosing errors, reconstitution errors, needle misuse, and lack of sterile technique. Public health guidance on injection safety emphasizes aseptic technique, sterile equipment, and avoidance of unsafe needle or syringe practices [38]. Injection also magnifies product-quality concerns because contaminated or misprepared products can bypass protective barriers and enter tissue or systemic circulation [17].

Pharmacological risk varies by compound, route, duration, user characteristics, and co-exposures. Peptides are biologically active signaling molecules, and their appeal often comes from their ability to influence pathways related to metabolism, appetite, tissue repair, inflammation, growth hormone release, pigmentation, libido, cognition, or immune function [1]. For many experimental peptides, the key issue is not that specific harms are definitively established, but that safety is insufficiently characterized in the populations, combinations, and durations being used by consumers. This is particularly relevant for compounds affecting endocrine or growth-factor pathways, where consumer use may overlap with performance enhancement, anti-doping concerns, and limited long-term safety evidence [18,32].

Regulatory ambiguity intensifies these risks. Peptides cannot be treated as a single legal or regulatory category: their status varies by compound, intended use, route of administration, manufacturing pathway, jurisdiction, indication, and access route. A peptide may be an approved medicine in one context, an investigational drug in another, a compounded product under specific conditions, a prohibited substance in sport, or an unapproved research chemical when sold online [11,18,21]. The "research use only" or "not for human consumption" label is especially problematic because it may shift liability away from vendors while products circulate in consumer environments where self-administration is discussed or anticipated [12]. In this context, regulatory risk depends less on the peptide name alone than on whether the product remains within an accountable clinical and supply-chain pathway [12].

Surveillance failure is a central public health concern. Current systems may not capture who is using nonprescribed peptides, what products are being used, where they are sourced, whether users are stacking compounds, or what adverse events occur. Acute harms such as injection-site infection may be visible, but chronic metabolic, endocrine, immune, reproductive, dermatologic, or oncologic signals may be harder to detect. When products are poorly studied, inconsistently labeled, or used in combination, adverse events may be difficult to interpret and report. These concerns align with broader evidence on adverse-event underreporting and attribution barriers, including in voluntary reporting systems and nonprescription product contexts [23,24]. Pharmacovigilance research on compounded GLP-1 receptor agonists further illustrates how nonstandard products can complicate adverse-event and medication-error reporting [34].

These risks raise broader ethical and equity concerns. Digital peptide promotion can blur the boundary between education, advertising, anecdote, and medical advice, especially when influencers, online clinicians, vendors, or affiliate marketers benefit from sales, consultations, sponsorships, referral links, or paid memberships [16,35]. Wealthier consumers may access peptides through private clinics with monitoring and higher-quality sourcing, while others rely on cheaper online vendors or informal suppliers. This creates stratified safety, where similar goals lead consumers into different risk environments depending on income, health literacy, geography, and access to trusted care.

Finally, supply-chain integrity has a biosecurity dimension. Most immediate risks are likely to involve accidental or commercial failures, including counterfeit products, mislabeling, contamination, inaccurate potency, unstable formulations, or poor manufacturing conditions [20,36]. However, the same opacity that enables low-quality products could also create theoretical vulnerabilities to deliberate adulteration or malicious substitution [20,22]. Direct evidence of deliberate biological harm through gray-market peptide products remains limited, but the normalization of self-injected products with uncertain provenance creates a broader supply-chain governance concern.

Together, these concerns show that unregulated peptide use is not simply individual risk-taking or isolated product misuse. It is a systems-level public health issue created by the convergence of uncertain evidence, persuasive digital promotion, fragmented regulation, opaque supply chains, experimental self-injection, and weak surveillance.

Research, Policy, and Clinical Priorities

The public health concerns surrounding unregulated peptide use require targeted research and proportionate policy and clinical responses. Current knowledge is fragmented across clinical pharmacology, toxicology, regulatory science, digital health, sports medicine, endocrinology, consumer behavior, and biosecurity. Recent digital and forum-based studies have begun to document how peptides are discussed, sourced, and interpreted by users online [15,33]. Regulatory and product-quality evidence also shows that online or nonstandard peptide-related products can raise concerns around identity, quality, safety, and oversight [21,36]. In many cases, public use appears to be moving faster than formal evidence generation, leaving clinicians, regulators, and public health agencies in a reactive position.

Research should first establish the prevalence and patterns of nonprescribed peptide use. Studies are needed to identify who is using these products, which peptides are being used, where they are obtained, whether they are taken alone or in combination, and what motivations drive use. Existing evidence from online peptide discussions and direct-to-consumer GLP-1 markets suggests that consumer access and self-experimentation are already occurring in digitally mediated settings [29,33]. Standardized exposure definitions are essential to distinguish approved prescription use, off-label clinical use, compounded products, investigational drugs, research-chemical products, and informal gray-market use.

Digital health research should examine how peptide demand is created and sustained online. Content analysis could assess how peptides are framed across YouTube, TikTok, Instagram, Reddit, Discord, podcasts, online clinics, newsletters, vendor websites, and private communities. Key questions include what benefits are claimed, what risks are disclosed, whether evidence is cited, whether conflicts of interest are transparent, and how approved medicines are presented alongside investigational or unapproved products. This is especially important because social media and online advertising studies show that digital platforms can shape public understanding of GLP-1 medicines and compounded products [10,35].

Product-quality and pharmacovigilance research should clarify what consumers are receiving and what harms may occur. Laboratory testing of online peptide products should assess identity, purity, potency, sterility, endotoxin contamination, degradation, solvent quality, and concordance with label claims. Product-quality testing of online semaglutide products illustrates why such market surveillance is needed [36]. Clinical research should characterize acute harms such as injection-site infections, allergic reactions, gastrointestinal symptoms, endocrine disturbances, dermatologic changes, metabolic abnormalities, and contamination-related illness. Longer-term studies are needed to assess endocrine, metabolic, immune, reproductive, dermatologic, oncologic, and interaction-related outcomes among people using peptides chronically or in combination with GLP-1 medications, testosterone, anabolic agents, supplements, or nootropics.

Policy responses should focus on monitoring, communication, and enforcement. Regulators and public health agencies should track direct-to-consumer websites, research-chemical vendors, online clinics, social media-linked sellers, and international suppliers, with attention to misleading claims, human-use marketing, hidden affiliate relationships, certificates of analysis used as reassurance, and "research use only" labels that may obscure consumer-directed use. FDA and Health Canada warnings already illustrate regulatory concern about unapproved GLP-1 products, unauthorized injectable peptides, and online access pathways [11,12]. Public-facing communication should clearly distinguish approved peptide medicines from compounded products, investigational drugs, research chemicals, counterfeit products, and prohibited substances in sport [18,21].

Policy messaging should avoid alarmism and should not imply that all peptides are unsafe. Instead, it should emphasize specific risks: uncertain identity, inaccurate potency, contamination, nonsterility, self-injection, stacking, lack of monitoring, and poor adverse-event capture. WHO guidance on substandard and falsified medical products supports the importance of prevention, detection, and response systems for poorly traceable medical products [20]. Supply-chain integrity should also be incorporated into policy thinking through traceability, batch documentation, product testing, and monitoring of online biological-product markets [22].

Although this review does not propose a clinical practice guideline, clinicians may still play an important surveillance role by approaching peptide use with curiosity rather than judgment. Peptide exposure should be incorporated into medication and supplement histories, especially in primary care, endocrinology, obesity medicine, sports medicine, dermatology, sexual health, emergency medicine, psychiatry, and men's health settings. Patients may not identify these products as medications, so clinicians may need to ask specifically about "peptides," "research compounds," "biohacking injections," "recovery peptides," "weight-loss injections," "longevity products," or "online injectable products." This recommendation is consistent with evidence that nonprescription or supplement-like exposures may be underreported or difficult for clinicians to capture [24].

When peptide use is disclosed, clinicians should document the product name, source, route, duration, reason for use, co-use with other agents, and adverse symptoms. Assessment should be guided by peptide type, route, and symptoms, with particular attention to injection-site infection, contamination-related illness, metabolic or endocrine abnormalities, dermatologic changes, and interactions with prescription drugs or enhancement-oriented products. For injectable products, basic injection-safety principles remain relevant because unsafe injection practices can increase infection and injury risk [38]. Suspected adverse events, medication errors, therapeutic failures, and product-quality problems should be reported through appropriate pharmacovigilance channels, including MedWatch in the United States, where applicable [34,39].

Harm-reduction principles remain important for patients who continue using peptides despite counseling. Encouraging disclosure, discouraging unknown sources, warning against needle sharing or nonsterile practices, advising against stacking multiple poorly studied compounds, and recommending early medical evaluation for concerning symptoms can reduce harm without endorsing unregulated use. The central aim should be proportionate prevention: protecting legitimate access to approved peptide medicines while reducing risks created by unapproved, poorly traceable, and digitally promoted peptide products.

## Conclusions

Peptide-based medicines have important and legitimate roles in modern clinical care, but their growing visibility has also contributed to a less regulated consumer marketplace where experimental or weakly evidenced products are promoted for optimization, aesthetics, performance, recovery, and longevity. The key public health concern is not peptide therapeutics themselves, but the normalization of poorly traceable peptide products used outside clinical, regulatory, and pharmacovigilance systems.

Public health responses should focus on clearer regulatory communication, clinician awareness, adverse-event reporting, product-quality monitoring, digital platform accountability, and harm-reduction strategies. These steps can help protect legitimate peptide medicine while reducing risks linked to gray-market access, self-injection, stacking, uncertain product quality, and incomplete surveillance.
